# Long-Term Survival With Pembrolizumab Re-administration After Pseudo-Progression With Immune-Related Interstitial Lung Disease in a Patient With Non-small Cell Lung Cancer

**DOI:** 10.7759/cureus.16177

**Published:** 2021-07-04

**Authors:** Koki Nakashima, Yoshiki Demura, Masaya Akai, Tamotsu Ishizuka

**Affiliations:** 1 Respiratory Medicine, University of Fukui Hospital, Fukui, JPN; 2 Respiratory Medicine, Japanese Red Cross Fukui Hospital, Fukui, JPN

**Keywords:** immune checkpoint inhibitor, lung cancer, pembrolizumab, pseudo progression, malignant pleural effusion

## Abstract

Immune checkpoint inhibitors may cause specific immune-related reactions, such as pseudo-progression. In particular, malignant pleural effusion tends to worsen due to this phenomenon. However, the appropriate management in such cases is unclear.

We report a 73-year-old man with advanced lung adenocarcinoma and malignant pleural effusion who developed pseudo-progression with immune-related interstitial lung disease (irILD) induced by pembrolizumab (Merck & Co., Kenilworth, NJ, USA). After managing them with steroid treatments and chemotherapy, pembrolizumab was re-administered. At the time of writing, 30 months have passed since the re-administration of pembrolizumab without disease progression. This clinical course conveys an appropriate management strategy for patients with pseudo-progression and irILD.

## Introduction

Immune checkpoint inhibitors (ICIs) are an effective treatment option for several types of cancers, including non-small cell lung cancer (NSCLC). However, ICIs may cause atypical patterns of response, including pseudo-progression [1,2]. When thoracic lesions worsen during ICI treatment, pseudo-progression, immune-related adverse events (irAEs), and disease progression including hyperprogressive disease (HPD) must be suspected. However, it is difficult for clinicians to correctly distinguish between them in clinical practice. Moreover, it may be possible that these phenomena coexist. Therefore, information about the appropriate management strategy in such cases is needed.

We herein report a 73-year-old man with NSCLC who developed pseudo-progression and immune-related interstitial lung disease (irILD) after initiation of treatment with ICI. However, steroid treatment dramatically reduced the tumor lesions and improved irILD. Therefore, we decided to re-administer pembrolizumab after second-line chemotherapy. Re-administration of pembrolizumab led to long-term survival, and the patient achieved a 30-month progression-free survival duration since the re-administration of pembrolizumab (35 months since diagnosis).

This case suggests that clinicians should consider the re-administration of ICIs in patients exhibiting pseudo-progression and irILD after management to achieve long-term survival.

## Case presentation

**Figure 1:**
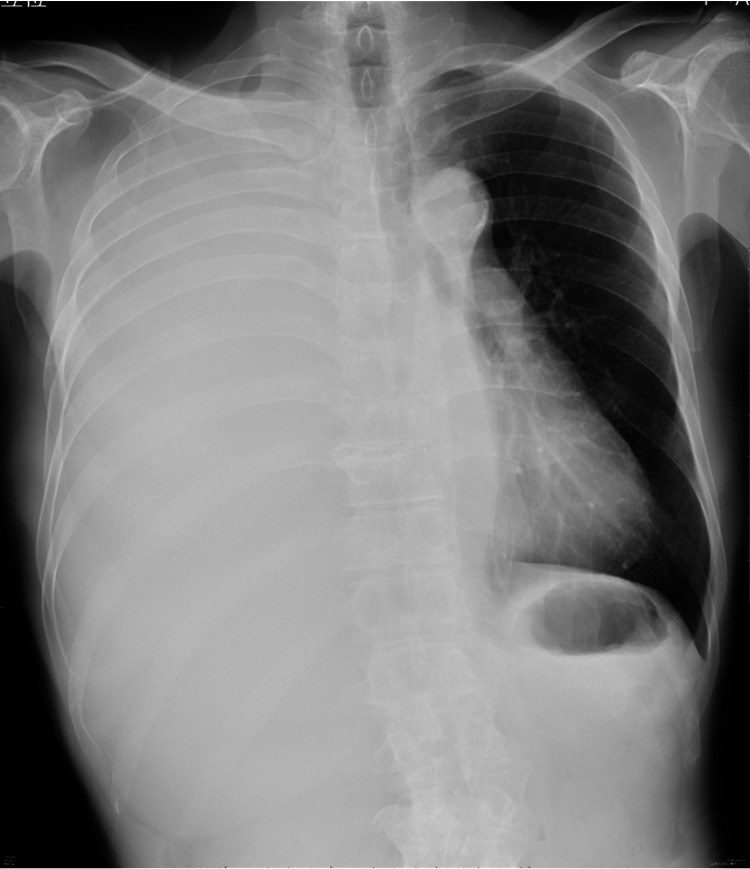
Chest X-ray at the first visit to our hospital Chest X-ray at the first visit to our hospital showed large amount of pleural effusion.

A 73-year-old man with a 50-year history of smoking (150 pack-year) presented to our hospital with right chest pain and dyspnea. Chest exam revealed decreased right breath sound. Chest radiography revealed a large amount of right pleural effusion (Figure 1).

**Figure 2:**
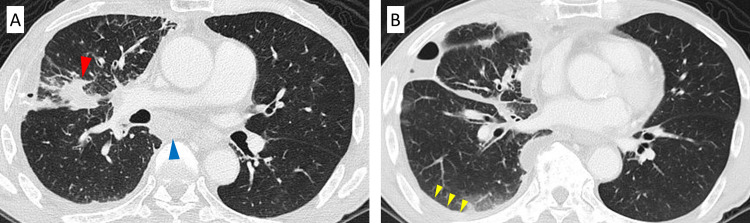
Chest computed tomography before the initiation of treatment with pembrolizumab Chest computed tomography before the initiation of treatment with pembrolizumab showed the primary tumor in right upper lung (red arrow), swelling of #7 lymph node (blue arrow) (A), and right mild pleural effusion (yellow arrows) (B).

Thoracoscopy under local anesthesia revealed a tumor in the pleura, and pathological findings led to the diagnosis of lung adenocarcinoma with a programmed cell death ligand-1 (PD-L1) tumor proportion score (TPS) of 95%. Driver oncogenes, including the epidermal growth factor receptor (EGFR), anaplastic lymphoma kinase (ALK) fusion gene, and c-ros oncogene 1 (ROS-1) fusion gene, were negative. Computed tomography (CT) and positron emission tomography with 18F-fluorodeoxyglucose after biopsy and thoracic drainage revealed primary tumor in the right middle lobe, hilar and mediastinal lymph nodes, right pleura, bone, liver, and right adrenal gland (Figure 2). Brain magnetic resonance imaging showed no findings suggesting central nervous system metastasis. These examination results led to the diagnosis of advanced lung adenocarcinoma (cT2aN3M1c; stage IVB).

**Figure 3:**
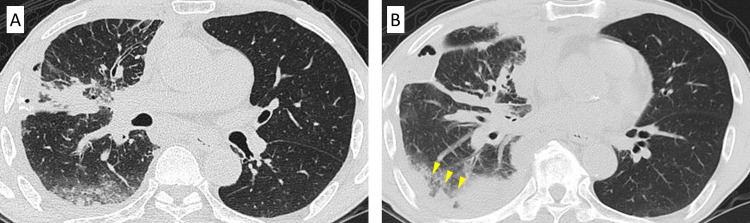
Chest computed tomography on day 7 after initiation of treatment with pembrolizumab Chest computed tomography on day 7 after initiation of treatment with pembrolizumab showed that pleural effusion worsened (yellow arrows).

**Figure 4:**
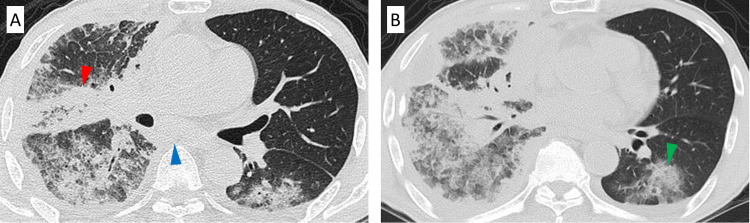
Chest computed tomography on day 17 after initiation of treatment with pembrolizumab Chest computed tomography on day 17 after treatment initiation with pembrolizumab showed that the tumor size had increased (red arrow and blue arrow) (A), the pleural effusion had worsened, and new ground-glass shadows appeared in the left lung (green arrow) (B).

**Figure 5:**
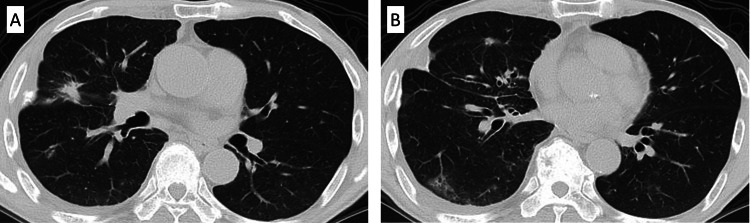
Chest computed tomography after a month since the initiation of steroid treatment Chest computed tomography after the initiation of steroid treatment showed improvements of solid lesions (A) and the pleural effusion, and the disappearance of the ground-glass shadows in both lungs (B).

After managing the right pleural effusion with intrathoracic administration of sterile graded talc, first-line chemotherapy with pembrolizumab was initiated. Although the right pleural effusion worsened after administering pembrolizumab, follow-up without any therapeutic interventions was continued because we suspected pseudo-progression (Figure 3). However, CT on day 17 revealed that the pleural effusion had worsened, the tumor size had increased, and new ground-glass shadows had appeared in both lungs (Figure 4). Based on these findings, we suspected irILD with pseudo-progression. The patient had mild dyspnea and an oxygen saturation level of 93% in room air, despite a baseline oxygen saturation level was 98%. Therefore, steroid treatment with methylprednisolone (500 mg/dL) was initiated for three days. CT after a month since initiation of steroid treatment showed that improvements in the pleural effusion and solid lesions and the disappearance of the ground-glass shadows in both lungs (Figure 5).

After reducing the prednisolone dose to 10 mg/day, second-line chemotherapy with carboplatin (AUC 4), pemetrexed (400 mg/m^2^) and bevacizumab (15 mg/kg) was administered every three weeks, resulting in stable disease. Steroid treatment was discontinued during second-line chemotherapy because the pleural effusion and ground-glass shadows improved. Thus, we decided to re-administrate pembrolizumab after four cycles of second-line chemotherapy to achieve long-term survival. The pleural effusion, ground-glass shadows and other tumor lesions did not worsen despite the re-administration of pembrolizumab. At present, the patient has achieved 30 months of survival after the re-administration of pembrolizumab (35 months since diagnosis), and chemotherapy with pembrolizumab is ongoing (41 cycles at the time of writing) without disease progression.

## Discussion

ICIs may cause atypical patterns of response, such as pseudo-progression, HPD, and irAEs. Furthermore, it may have possible that these phenomena coexist. In several clinical trials of ICIs in patients with NSCLC, patients treated with ICIs had poorer outcomes than those of patients treated with cytotoxic chemotherapy within three to six months after treatment initiation [3,4]. These results indicate that early immune-related reactions, such as pseudo-progression, HPD, or irAEs, might be a cause of poor outcomes in the early stage of the disease. In fact, several reports have suggested that life-threatening immune-related reactions are caused by the initiation of ICI treatment in NSCLC patients with serositis [5,6]. However, it is unclear that ICI should be re-administrated for such cases.

We considered the worsening of the thoracic lesions as a manifestation of pseudo-progression at first, because the reaction mainly occurred at the tumor lesion. However, ground-glass shadows appeared in the left lung as new lesions could not be considered pseudo-progression, because there was no tumor lesion. Therefore, we considered ground-glass shadows as irILD, and decided to initiate treatment with steroid. 

The administration of four cycles of cytotoxic chemotherapy with carboplatin, pemetrexed, and bevacizumab, as well as steroids, before the re-administration of ICI is one of the factors of successful re-administration of ICIs. Several reports have indicated that patients with a large tumor size have poorer survival outcomes than those of patients with a small tumor size [7,8]. These results suggest that a small tumor size is an ideal situation for ICIs. Therefore, we administered four cycles of cytotoxic chemotherapy to decrease the tumor size and control disease activity. Moreover, we confirmed that steroid treatment could be discontinued until four cycles of cytotoxic chemotherapy without disease progression. Therefore, we decided to re-administer pembrolizumab after four cycles of cytotoxic chemotherapy. 

The clinical course in our patient suggests two important clinical issues. First, steroid treatment is effective to reduce the activity of pseudo-progression, as well as irILD. The mechanism of pseudo-progression involves infiltration and the recruitment of immune cells, such as lymphocytes, into tumors [1,9]. In general, immunosuppressive drugs might be better used only for cases of life-threatening immune-related reactions, because immunosuppressive drugs can decrease ICI effects [10]. However, steroid treatment may be an effective treatment option in cases of strong pseudo-progression with irILD, as in the present case.

Second, long-term survival may be achieved with ICI re-administration after the management of strong immune-related reactions, even if a steroid is administered. Arbour et al. reported that steroid treatment might decrease the efficacy of ICIs [10]. Thus, clinicians tend to avoid administering immunosuppressive drugs during ICI treatment. However, immunosuppressive treatments are needed in cases of strong immune-related reactions, as in the present case. In such cases, short-term immunosuppressive treatment should be administered to elicit the effects of ICIs. In fact, the patient in the present case achieved long-term survival, although steroid treatment was administered for approximately two months.

Several studies have demonstrated that ICI re-challenge after disease progression with initial ICIs is effective in some patients, although this effect is limited [11,12]. Furthermore, Dolladille et al. also indicated that the rate of irAE recurrence with ICI re-challenge was approximately 30% in patients who developed irAEs with initial ICI treatment [13]. These results suggest that ICI re-challenge should be considered in appropriate cases. Our case indicates that ICI re-challenge might be effective in patients with strong immune-related reactions after management.

It is challenging to manage immune-related reactions and re-administrate ICI. However, if clinicians avoid re-administering ICI after immune-related reactions completely, patients may forgo opportunities for long-term survival with ICI therapy. In fact, Won et al. indicated that patients with pseudo-progression show significantly better outcomes than those of patients without pseudo-progression [14]. Furthermore, Teraoka et al. indicated that early irAEs are associated with a better outcome after treatment with ICI [15]. Therefore, clinicians should consider re-administering ICI after management of immune-related reactions.

## Conclusions

In conclusion, we herein described a case of long-term survival with the re-administration of pembrolizumab after managing pseudo-progression with irILD, which represented worsening pleural effusion and the new appearance of ground-glass shadows. We recommend that ICI re-administration should be considered to achieve long-term survival if immune-related reactions are manageable.
